# The genome sequence of the ten-spot ladybird, *Adalia decempunctata *(Linnaeus, 1758)

**DOI:** 10.12688/wellcomeopenres.21008.1

**Published:** 2024-03-01

**Authors:** Liam M. Crowley, Helen E. Roy, Peter M.J. Brown, University of Oxford and Wytham Woods Genome Acquisition Lab, Darwin Tree of Life Barcoding collective, Wellcome Sanger Institute Tree of Life Management, Samples and Laboratory team, Wellcome Sanger Institute Scientific Operations: Sequencing Operations, Wellcome Sanger Institute Tree of Life Core Informatics team, Tree of Life Core Informatics collective, Darwin Tree of Life Consortium

**Affiliations:** 1 University of Oxford, Oxford, England, UK; 2 UK Centre for Ecology & Hydrology, Crowmarsh Gifford, England, UK; 3 Anglia Ruskin University, Cambridge, England, UK

**Keywords:** Adalia decempunctata, ten-spot ladybird, genome sequence, chromosomal, Coleoptera

## Abstract

We present a genome assembly from an individual male *Adalia decempunctata* (the ten-spot ladybird; Arthropoda; Insecta; Coleoptera; Coccinellidae). The genome sequence is 489.4 megabases in span. Most of the assembly is scaffolded into 12 chromosomal pseudomolecules, including the X and Y sex chromosomes. The mitochondrial genome has also been assembled and is 19.68 kilobases in length.

## Species taxonomy

Eukaryota; Opisthokonta; Metazoa; Eumetazoa; Bilateria; Protostomia; Ecdysozoa; Panarthropoda; Arthropoda; Mandibulata; Pancrustacea; Hexapoda; Insecta; Dicondylia; Pterygota; Neoptera; Endopterygota; Coleoptera; Polyphaga; Cucujiformia; Coccinelloidea; Coccinellidae; Coccinellinae; Coccinellini; *Adalia*; *Adalia decempunctata* (Linnaeus, 1758) (NCBI:txid115343).

## Background

The ten-spot ladybird, *Adalia decempunctata*, is medium sized (3.5–5.0 mm) conspicuously marked ladybird. It is predominantly a western Palearctic species and is locally common across the UK. The colour patterns of the elytra are highly variable and in the UK there are three common forms: orange with up to 15 dark spots (typical form or f. *decempunctata*), brown or black with a light grid-like marking (chequered form or f. *decempustulatus*) and a very dark form with yellow, orange or red shoulder flashes (melanic form or f. *bimaculata*). As for most ladybirds, the contrasting colours and strong patterns are considered to be warning colouration, and so it is puzzling that there is so much variation because evolutionary theory might suggest that displaying one colour pattern would be more consistent as a message for potential predators.

A recent study has highlighted that ten-spot ladybirds are not only morphologically variable but that the mitochondrial *COI* gene is highly variable too, eight different haplotypes were found from a sample of 92 individuals from across Europe ( Shaikevich *et al.*, 2019). Different strains of the male-killing *Rickettsia* bacterium infecting *A. bipunctata* have been shown to be associated with distinct mitochondrial haplotypes. This is perhaps unsurprising since both the bacterium and mitochondria are maternally transmitted ( Jiggins & Tinsley, 2005). However, this is yet to be proven for *A. decempunctata* which seems to have a low prevalence of *Rickettsia* infection ( Shaikevich *et al.*, 2019).

It is univoltine in the UK, although on the continent it is more frequently bivoltine. Adults are active from March, mating from April to May and oviposition continuing into June. The predaceous larvae emerge after a few days and feed on aphids and other soft bodied insects throughout the summer, undergoing 4 instars before pupating around July to August. Newly eclosed adults continue to feed into the autumn months before overwintering. Although it is mostly a diurnal species, it is well known to be attracted to light and frequently comes to light traps.

Ten-spot ladybirds are close relatives of the two-spot ladybirds, *Adalia bipunctata* ( Wellcome Sanger Institute Tree of Life programme *et al.*, 2022), and occupy similar habitats with both particularly favouring deciduous trees and hedgerows, although the ten-spot is perhaps more arboreal. The harlequin ladybird, *Harmonia axyridis* ( Boyes *et al.*, 2021), an invasive non-native species in the UK ( Roy *et al.*, 2016), also favours these habitats. The harlequin ladybird is a much larger and more voracious than both the two-spot and ten-spot ladybird. Both species are experiencing strong declines in distributions correlated with the arrival of the harlequin ladybird. However, the decline of the ten-spot ladybird is less pronounced than that of the two-spot ladybird. Interestingly, ten-spot ladybirds have recently been reported as a non-native species having established in Canada ( Langor *et al.*, 2023).

The genome of *Adalia decempunctata* was sequenced as part of the Darwin Tree of Life Project, a collaborative effort to sequence all named eukaryotic species in the Atlantic Archipelago of Britain and Ireland. Here we present a chromosomally complete genome sequence for *Adalia decempunctata*, based on one *Adalia decempunctata* specimen from Wytham Woods, Oxfordshire, UK.

## Genome sequence report

**Figure 1.**
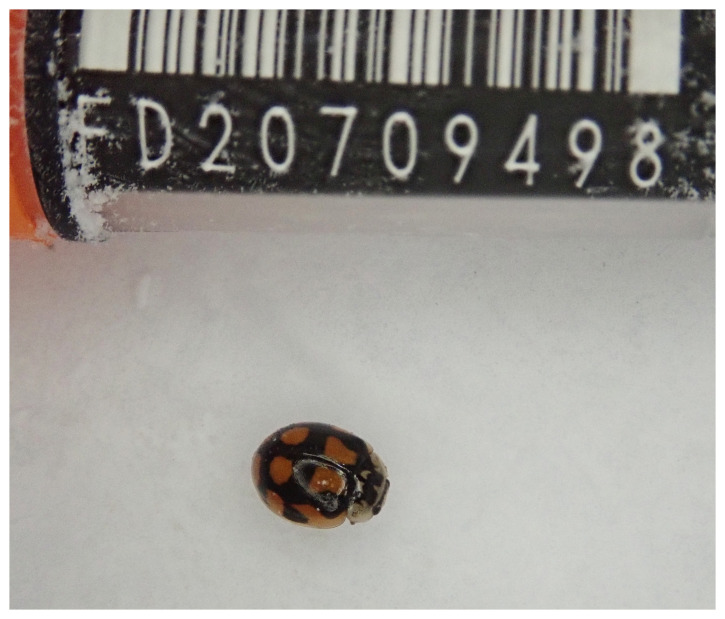
Photograph of the *Adalia decempunctata* (icAdaDece3) specimen used for genome sequencing.

The genome was sequenced from one male *Adalia decempunctata* ( Figure 1) collected from Wytham Woods, Oxfordshire, UK (51.77, –1.31). A total of 46-fold coverage in Pacific Biosciences single-molecule HiFi long reads was generated. Primary assembly contigs were scaffolded with chromosome conformation Hi-C data. Manual assembly curation corrected 23 missing joins or mis-joins and removed 7 haplotypic duplications, reducing the assembly length by 1.28% and the scaffold number by 1.20%, and decreasing the scaffold N50 by 0.99%.

**Figure 2.**
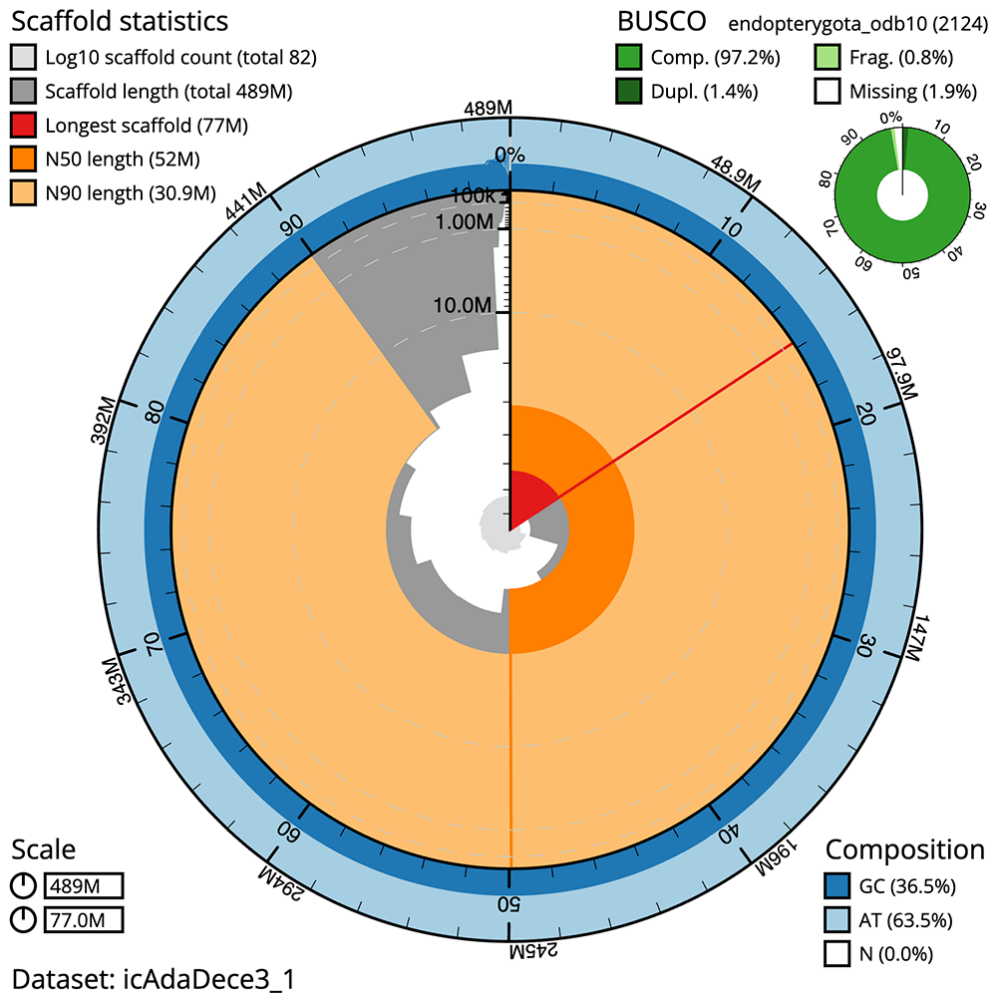
Genome assembly of *Adalia decempunctata*, icAdaDece3.1: metrics. The BlobToolKit Snailplot shows N50 metrics and BUSCO gene completeness. The main plot is divided into 1,000 size-ordered bins around the circumference with each bin representing 0.1% of the 489,453,138 bp assembly. The distribution of scaffold lengths is shown in dark grey with the plot radius scaled to the longest scaffold present in the assembly (76,995,679 bp, shown in red). Orange and pale-orange arcs show the N50 and N90 scaffold lengths (51,987,084 and 30,888,688 bp), respectively. The pale grey spiral shows the cumulative scaffold count on a log scale with white scale lines showing successive orders of magnitude. The blue and pale-blue area around the outside of the plot shows the distribution of GC, AT and N percentages in the same bins as the inner plot. A summary of complete, fragmented, duplicated and missing BUSCO genes in the endopterygota_odb10 set is shown in the top right. interactive version of this figure is available at https://blobtoolkit.genomehubs.org/view/icAdaDece3_1/dataset/icAdaDece3_1/snail.

**Figure 3.**
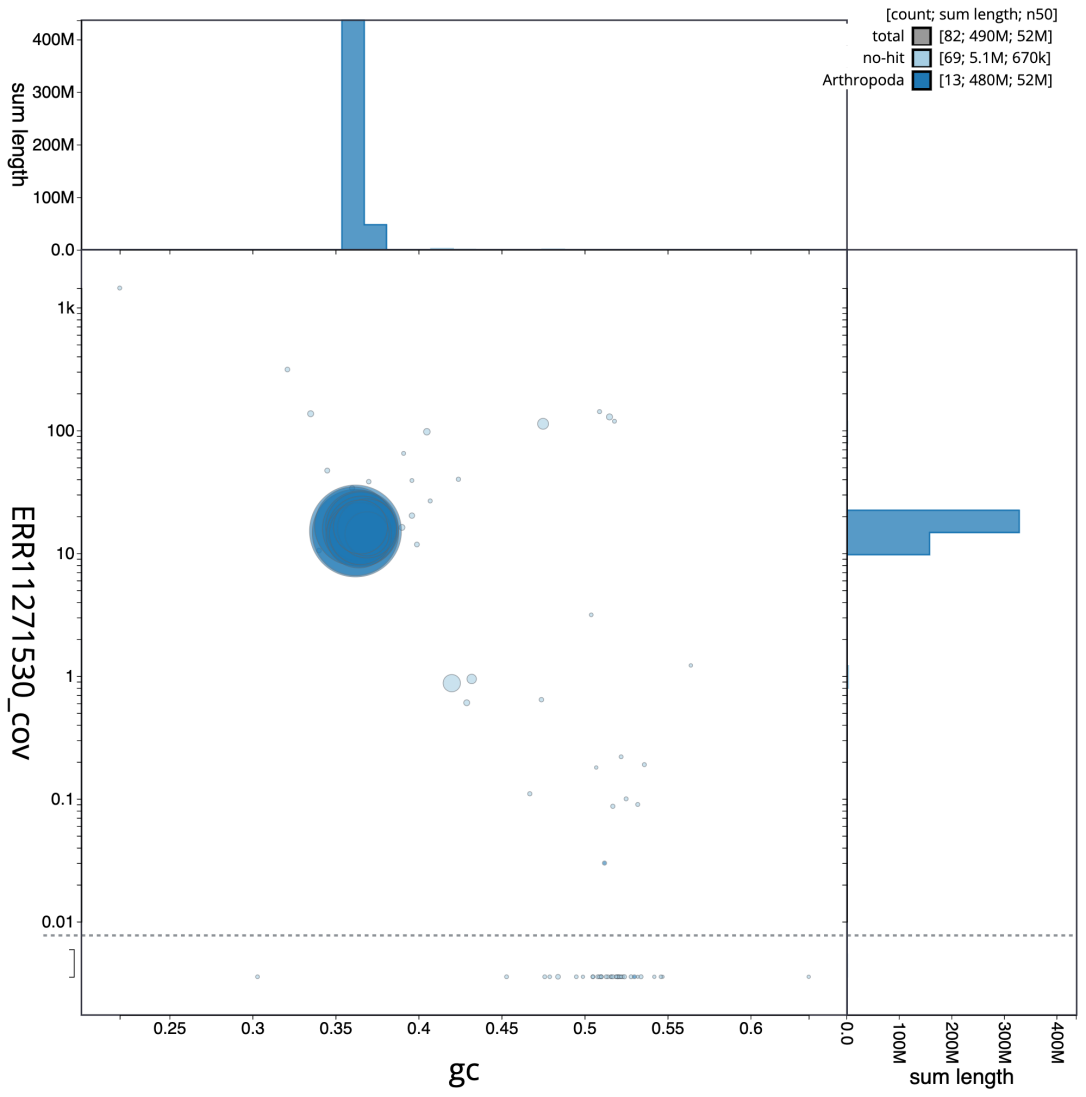
Genome assembly of *Adalia decempunctata*, icAdaDece3.1: BlobToolKit GC-coverage plot. Scaffolds are coloured by phylum. Circles are sized in proportion to scaffold length. Histograms show the distribution of scaffold length sum along each axis. An interactive version of this figure is available at https://blobtoolkit.genomehubs.org/view/icAdaDece3_1/dataset/icAdaDece3_1/blob.

**Figure 4.**
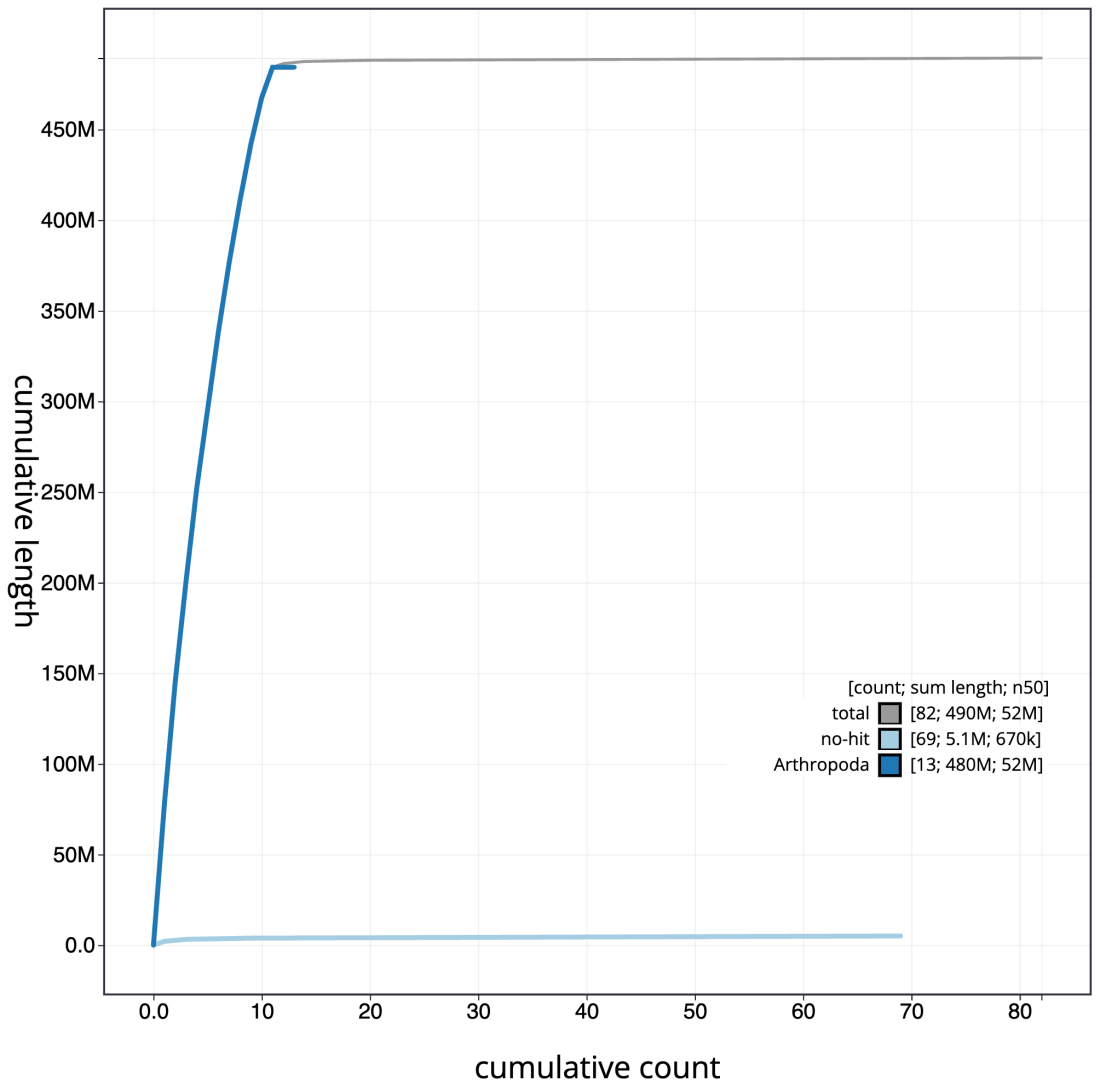
Genome assembly of *Adalia decempunctata*, icAdaDece3.1: BlobToolKit cumulative sequence plot. The grey line shows cumulative length for all scaffolds. Coloured lines show cumulative lengths of scaffolds assigned to each phylum using the buscogenes taxrule. An interactive version of this figure is available at https://blobtoolkit.genomehubs.org/view/icAdaDece3_1/dataset/icAdaDece3_1/cumulative.

**Figure 5.**
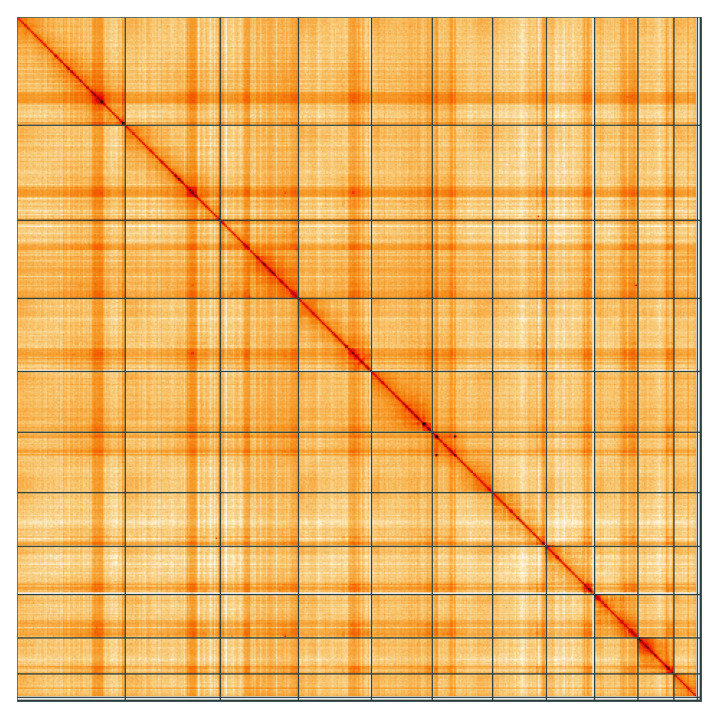
Genome assembly of *Adalia decempunctata*, icAdaDece3.1: Hi-C contact map of the icAdaDece3.1 assembly, visualised using HiGlass. Chromosomes are shown in order of size from left to right and top to bottom. An interactive version of this figure may be viewed at https://genome-note-higlass.tol.sanger.ac.uk/l/?d=Od9wibQtQU-zuj0tP1vPDw.

The final assembly has a total length of 489.4 Mb in 81 sequence scaffolds with a scaffold N50 of 52.0 Mb ( Table 1). The snailplot in Figure 2 provides a summary of the assembly statistics, while the distribution of assembly scaffolds on GC proportion and coverage is shown in Figure 3. The cumulative assembly plot in Figure 4 shows curves for subsets of scaffolds assigned to different phyla. Most (99.63%) of the assembly sequence was assigned to 12 chromosomal-level scaffolds, representing 10 autosomes and the X and Y sex chromosomes. Chromosome-scale scaffolds confirmed by the Hi-C data are named in order of size ( Figure 5; Table 2). The assignment of sex chromosomes was guided by coverage statistics and synteny to *Adalia bipunctata* (GCA_910592335.1) ( Wellcome Sanger Institute Tree of Life programme *et al.*, 2022). While not fully phased, the assembly deposited is of one haplotype. Contigs corresponding to the second haplotype have also been deposited. The mitochondrial genome was also assembled and can be found as a contig within the multifasta file of the genome submission.

**Table 1.** Genome data for *Adalia decempunctata*, icAdaDece3.1.

Project accession data		
Assembly identifier	icAdaDece3.1	
Species	*Adalia decempunctata*	
Specimen	icAdaDece3	
NCBI taxonomy ID	115343	
BioProject	PRJEB61609	
BioSample ID	SAMEA10107051	
Isolate information	icAdaDece3, male: whole organism (DNA sequencing) icAdaDece1: whole organism (Hi-C sequencing)	
Assembly metrics *		*Benchmark*
Consensus quality (QV)	66.5	*≥ 50*
*k*-mer completeness	100.0%	*≥ 95%*
BUSCO **	C:97.2%[S:95.9%,D:1.4%], F:0.8%,M:1.9%,n:2,124	*C ≥ 95%*
Percentage of assembly mapped to chromosomes	99.63%	*≥ 95%*
Sex chromosomes	XY	*localised * *homologous pairs*
Organelles	Mitochondrial genome: 19.68 kb	*complete single * *alleles*
Raw data accessions		
PacificBiosciences SEQUEL II	ERR11279085, ERR11279086	
Hi-C Illumina	ERR11271526	
Genome assembly		
Assembly accession	GCA_951802165.1	
*Accession of alternate haplotype*	GCA_951802185.1	
Span (Mb)	489.4	
Number of contigs	130	
Contig N50 length (Mb)	26.4	
Number of scaffolds	81	
Scaffold N50 length (Mb)	52.0	
Longest scaffold (Mb)	77.0	

* Assembly metric benchmarks are adapted from column VGP-2020 of “Table 1: Proposed standards and metrics for defining genome assembly quality” from ( Rhie *et al.*, 2021). ** BUSCO scores based on the endopterygota_odb10 BUSCO set using version 5.3.2. C = complete [S = single copy, D = duplicated], F = fragmented, M = missing, n = number of orthologues in comparison. A full set of BUSCO scores is available at https://blobtoolkit.genomehubs.org/view/icAdaDece3_1/dataset/icAdaDece3_1/busco.

**Table 2.** Chromosomal pseudomolecules in the genome assembly of *Adalia decempunctata*, icAdaDece3.

INSDC accession	Chromosome	Length (Mb)	GC%
OX637695.1	1	77.0	36.0
OX637696.1	2	67.69	36.0
OX637697.1	3	55.62	36.0
OX637698.1	4	51.99	36.5
OX637699.1	5	43.29	36.5
OX637700.1	6	43.02	36.5
OX637701.1	7	38.22	36.5
OX637702.1	8	34.32	36.5
OX637703.1	9	30.89	37.0
OX637705.1	10	16.71	37.0
OX637704.1	X	25.61	36.5
OX637706.1	Y	2.09	42.0
OX637707.1	MT	0.02	22.0

The estimated Quality Value (QV) of the final assembly is 66.5 with *k*-mer completeness of 100.0%, and the assembly has a BUSCO v5.3.2 completeness of 97.2% (single = 95.9%, duplicated = 1.4%), using the endopterygota_odb10 reference set ( *n* = 2,124).

Metadata for specimens, barcode results, spectra estimates, sequencing runs, contaminants and pre-curation assembly statistics are given at https://links.tol.sanger.ac.uk/species/115343.

## Methods

### Sample acquisition and nucleic acid extraction

The specimen used for genome sequencing was a male *Adalia decempunctata* (specimen ID Ox001130, ToLID icAdaDece3), which was potted in Wytham Woods, Oxfordshire (biological vice-county Berkshire), UK (latitude 51.77, longitude –1.31) on 2021-04-19. The specimen used for Hi-C sequencing (specimen ID Ox000328, ToLID icAdaDece1) was collected from the same location on 2020-01-08 using a light trap. The specimens were collected and identified by Liam Crowley (University of Oxford) and preserved on dry ice.

The workflow for high molecular weight (HMW) DNA extraction at the Wellcome Sanger Institute (WSI) includes a sequence of core procedures: sample preparation; sample homogenisation, DNA extraction, fragmentation, and clean-up. In sample preparation, the icAdaDece3 sample was weighed and dissected on dry ice ( Jay *et al.*, 2023). Tissue from the whole organism was homogenised using a PowerMasher II tissue disruptor ( Denton *et al.*, 2023a). HMW DNA was extracted using the Automated MagAttract v2 protocol ( Oatley *et al.*, 2023a). DNA was sheared into an average fragment size of 12–20 kb in a Megaruptor 3 system with speed setting 31 ( Bates *et al.*, 2023). Sheared DNA was purified by solid-phase reversible immobilisation ( Oatley *et al.*, 2023b): in brief, the method employs a 1.8X ratio of AMPure PB beads to sample to eliminate shorter fragments and concentrate the DNA. The concentration of the sheared and purified DNA was assessed using a Nanodrop spectrophotometer and Qubit Fluorometer and Qubit dsDNA High Sensitivity Assay kit. Fragment size distribution was evaluated by running the sample on the FemtoPulse system.

Protocols developed by the WSI Tree of Life laboratory are publicly available on protocols.io ( Denton *et al.*, 2023b).

### Sequencing

Pacific Biosciences HiFi circular consensus DNA sequencing libraries were constructed according to the manufacturers’ instructions. DNA sequencing was performed by the Scientific Operations core at the WSI on a Pacific Biosciences SEQUEL II instrument. Hi-C data were also generated from whole organism tissue of icAdaDece1 using the Arima2 kit and sequenced on the Illumina NovaSeq 6000 instrument.

### Genome assembly, curation and evaluation

Assembly was carried out with Hifiasm ( Cheng *et al.*, 2021) and haplotypic duplication was identified and removed with purge_dups ( Guan *et al.*, 2020). The assembly was then scaffolded with Hi-C data ( Rao *et al.*, 2014) using YaHS ( Zhou *et al.*, 2023). The assembly was checked for contamination and corrected as described previously ( Howe *et al.*, 2021). Manual curation was performed using HiGlass ( Kerpedjiev *et al.*, 2018) and PretextView ( Harry, 2022). The mitochondrial genome was assembled using MitoHiFi ( Uliano-Silva *et al.*, 2023), which runs MitoFinder ( Allio *et al.*, 2020) or MITOS ( Bernt *et al.*, 2013) and uses these annotations to select the final mitochondrial contig and to ensure the general quality of the sequence.

A Hi-C map for the final assembly was produced using bwa-mem2 ( Vasimuddin *et al.*, 2019) in the Cooler file format ( Abdennur & Mirny, 2020). To assess the assembly metrics, the *k*-mer completeness and QV consensus quality values were calculated in Merqury ( Rhie *et al.*, 2020). This work was done using Nextflow ( Di Tommaso *et al.*, 2017) DSL2 pipelines “sanger-tol/readmapping” ( Surana *et al.*, 2023a) and “sanger-tol/genomenote” ( Surana *et al.*, 2023b). The genome was analysed within the BlobToolKit environment ( Challis *et al.*, 2020) and BUSCO scores ( Manni *et al.*, 2021; Simão *et al.*, 2015) were calculated.

Table 3 contains a list of relevant software tool versions and sources.

**Table 3.** Software tools: versions and sources.

Software tool	Version	Source
BlobToolKit	4.2.0	https://github.com/blobtoolkit/blobtoolkit
BUSCO	5.3.2	https://gitlab.com/ezlab/busco
Hifiasm	0.16.1-r375	https://github.com/chhylp123/hifiasm
HiGlass	1.11.6	https://github.com/higlass/higlass
Merqury	MerquryFK	https://github.com/thegenemyers/MERQURY.FK
MitoHiFi	3	https://github.com/marcelauliano/MitoHiFi
PretextView	0.2	https://github.com/wtsi-hpag/PretextView
purge_dups	1.2.5	https://github.com/dfguan/purge_dups
sanger-tol/genomenote	v1.0	https://github.com/sanger-tol/genomenote
sanger-tol/readmapping	1.1.0	https://github.com/sanger-tol/readmapping/tree/1.1.0
YaHS	1.2a.2	https://github.com/c-zhou/yahs

### Wellcome Sanger Institute – Legal and Governance

The materials that have contributed to this genome note have been supplied by a Darwin Tree of Life Partner. The submission of materials by a Darwin Tree of Life Partner is subject to the ‘ **Darwin Tree of Life Project Sampling Code of Practice**’, which can be found in full on the Darwin Tree of Life website here. By agreeing with and signing up to the Sampling Code of Practice, the Darwin Tree of Life Partner agrees they will meet the legal and ethical requirements and standards set out within this document in respect of all samples acquired for, and supplied to, the Darwin Tree of Life Project.

Further, the Wellcome Sanger Institute employs a process whereby due diligence is carried out proportionate to the nature of the materials themselves, and the circumstances under which they have been/are to be collected and provided for use. The purpose of this is to address and mitigate any potential legal and/or ethical implications of receipt and use of the materials as part of the research project, and to ensure that in doing so we align with best practice wherever possible. The overarching areas of consideration are:

•       Ethical review of provenance and sourcing of the material

•       Legality of collection, transfer and use (national and international) 

Each transfer of samples is further undertaken according to a Research Collaboration Agreement or Material Transfer Agreement entered into by the Darwin Tree of Life Partner, Genome Research Limited (operating as the Wellcome Sanger Institute), and in some circumstances other Darwin Tree of Life collaborators.

## Data Availability

European Nucleotide Archive: *Adalia decempunctata* (10-spot ladybird). Accession number PRJEB61609; https://identifiers.org/ena.embl/PRJEB61609 ( Wellcome Sanger Institute, 2023). The genome sequence is released openly for reuse. The *Adalia decempunctata* genome sequencing initiative is part of the Darwin Tree of Life (DToL) project. All raw sequence data and the assembly have been deposited in INSDC databases. The genome will be annotated using available RNA-Seq data and presented through the Ensembl pipeline at the European Bioinformatics Institute. Raw data and assembly accession identifiers are reported in Table 1.

## Author information

Members of the University of Oxford and Wytham Woods Genome Acquisition Lab are listed here: https://doi.org/10.5281/zenodo.7125292.

Members of the Darwin Tree of Life Barcoding collective are listed here: https://doi.org/10.5281/zenodo.4893703.

Members of the Wellcome Sanger Institute Tree of Life Management, Samples and Laboratory team are listed here: https://doi.org/10.5281/zenodo.10066175.

Members of Wellcome Sanger Institute Scientific Operations: Sequencing Operations are listed here: https://doi.org/10.5281/zenodo.10043364.

Members of the Wellcome Sanger Institute Tree of Life Core Informatics team are listed here: https://doi.org/10.5281/zenodo.10066637.

Members of the Tree of Life Core Informatics collective are listed here: https://doi.org/10.5281/zenodo.5013541.

Members of the Darwin Tree of Life Consortium are listed here: https://doi.org/10.5281/zenodo.4783558.
