# Supplementary material for: The genome sequence of the ten-spot ladybird, Adalia decempunctata (Linnaeus, 1758)
Source: Wellcome Open Res. 2024 Mar 1;9:106. [Version 1] doi: 10.12688/wellcomeopenres.21008.1 (PMC11222777; doi:10.12688/wellcomeopenres.21008.1)
Supplement: Peer review history

# Reviewer report: Reviewer response for version 1

**DOI:** 10.21956/wellcomeopenres.23243.r86797

**Published:** 2024-08-05

**Authors:** Emily Hornett

**Affiliations:** 1 ​University of Liverpool, Liverpool, UK

In this data note the authors present a chromosome-level genome of the 10-spot ladybird, *Adalia decempunctata*. The rationale for this project is to add to the growing number of genomes of eukaryotic species sequenced by the Darwin Tree of Life Project.

 The methods and techniques are standard and sound. The writing is clear and concise.

**A few minor comments:**

- First line of Background - insert an “a” between “is” and “medium”.Methods …“which was potted”… - do you mean potted as in put into a pot, or do you actually mean “spotted”? Unusual phrasing if “potted”.
- Note the colour pattern of the sequenced individual in the methods (chequered going by the photo) - seeing as elytra colour was mentioned in the Background section.
- I do wonder whether the authors checked for the missing genes from BUSCO manually (using BLAST or similar) - they can sometimes be found in this manner, increasing completeness.

Are sufficient details of methods and materials provided to allow replication by others?

Partly

Is the rationale for creating the dataset(s) clearly described?

Yes

Are the datasets clearly presented in a useable and accessible format?

Yes

Are the protocols appropriate and is the work technically sound?

Yes

Reviewer Expertise:

Insect and bacterial genomics, evolutionary genetics, symbiosis, microbiomes.

I confirm that I have read this submission and believe that I have an appropriate level of expertise to confirm that it is of an acceptable scientific standard.

# Reviewer report: Reviewer response for version 1

**DOI:** 10.21956/wellcomeopenres.23243.r86796

**Published:** 2024-07-03

**Authors:** Terrence Sylvester

**Affiliations:** 1 The University of Memphis, Memphis, Tennessee, USA

The authors present a high-quality genome sequence of the ten-spot ladybird. The authors provide sufficient background on the beetle and the rationale for creating the dataset is clearly described. The manuscript follows a standard, widely accepted genome sequencing and assembly approach. The authors provide sufficient details on methods (including the programs used and versions) for replication of this study by others. Data are provided in a usable and accessible format. I do not have significant remarks related to the work presented in this manuscript.

Are sufficient details of methods and materials provided to allow replication by others?

Yes

Is the rationale for creating the dataset(s) clearly described?

Yes

Are the datasets clearly presented in a useable and accessible format?

Yes

Are the protocols appropriate and is the work technically sound?

Yes

Reviewer Expertise:

de-novo genomics

I confirm that I have read this submission and believe that I have an appropriate level of expertise to confirm that it is of an acceptable scientific standard.

# Reviewer report: Reviewer response for version 1

**DOI:** 10.21956/wellcomeopenres.23243.r86792

**Published:** 2024-07-03

**Authors:** Manee M Manee

**Affiliations:** 1 King Abdulaziz City for Science and Technology, Riyadh, Saudi Arabia

The study presents a high-quality genome assembly that provides significant insights into the genetic makeup of this species and contributes to the fields of entomology and genomics. The genome assembly of *Adalia decempunctata* serves as a valuable resource for future research on ladybird genetics, evolution, and ecology. Although the genome assembly is highly complete, the study could benefit from more extensive functional annotation and analysis of genetic features. Detailed annotation would provide deeper insights into the biological functions and evolutionary adaptations of the ten-spot ladybird.

Are sufficient details of methods and materials provided to allow replication by others?

Yes

Is the rationale for creating the dataset(s) clearly described?

Yes

Are the datasets clearly presented in a useable and accessible format?

Yes

Are the protocols appropriate and is the work technically sound?

Yes

Reviewer Expertise:

bioinformatics, genomics, and evolutionary biology

I confirm that I have read this submission and believe that I have an appropriate level of expertise to confirm that it is of an acceptable scientific standard.

# Reviewer report: Reviewer response for version 1

**DOI:** 10.21956/wellcomeopenres.23243.r86795

**Published:** 2024-06-19

**Authors:** Doga Cedden

**Affiliations:** 1 Department of Evolutionary Developmental Genetics, , GZMB, University of Göttingen, , Germany., Johann-Friedrich-Blumenbach Institute, Göttingen, Germany

The data note by Crowley et al. presents a high-quality genome assembly from the ten-spot ladybird. The manuscript contains sufficient background information about the beetle. The method is appropriate, and datasets are deposited in an accessible format. The authors also provide sufficient quality analysis of the genome assembly. I do not have any suggestions.

Are sufficient details of methods and materials provided to allow replication by others?

Yes

Is the rationale for creating the dataset(s) clearly described?

Yes

Are the datasets clearly presented in a useable and accessible format?

Yes

Are the protocols appropriate and is the work technically sound?

Yes

Reviewer Expertise:

I work on RNA interference and insect physiology in coleopteran pests.

I confirm that I have read this submission and believe that I have an appropriate level of expertise to confirm that it is of an acceptable scientific standard.
